# Impact of polypharmacy on inducing blood dyscrasias in clozapine treated patients

**DOI:** 10.1192/j.eurpsy.2022.1855

**Published:** 2022-09-01

**Authors:** M. Shiri, A. Ouertani, U. Ouali, R. Jomli

**Affiliations:** 1 Razi hospital, Psychiatry A, Manouba, Tunisia; 2 Razi Hospital, Psychiatry A, manouba, Tunisia

**Keywords:** clozapine, psychotropic medication, Polypharmacy, blood dyscrasias

## Abstract

**Introduction:**

Clozapine is commonly associated with hematological side effects. However, little research is available on the impact of adding other psychotropic medication on inducing blood dyscrasias.

**Objectives:**

The aim of the study was to explore the impact of associating psychotropic medication to clozapine in producing hematological abnormalities.

**Methods:**

Our study was a longitudinal, retrospective chart review of adult psychiatric patients receiving clozapine treatment at our clozapine consultation between January 2000 and September 2020.

**Results:**

Our sample consisted of 15 women (23.5%) and 49 men (76.5%), mean age was 41.34 ±9.32 years. Polypharmacy was found in 70.3% of the cases. Association of clozapine to other psychotropic agents was found in 67.2% of the cases. Most prescribed add-on medication was valproic acid in 27 cases, benzodiazepines in 21 cases, promethazine and hydroxyzine in 16 cases, lithium in 8 cases and haloperidol in 6 cases. We found blood dyscrasias in 21 patients (32.8%). Hematological abnormalities were as follow: 2 cases of agranulocytosis, 8 cases of neutropenia, 13 cases of thrombocytopenia, 5 cases of leukocytosis, 5 cases of eosinophilia and 3 cases of anemia. In our sample we did not find a significant association between psychotropic polypharmacy and blood dyscrasias.

**Conclusions:**

Many psychiatric patients on clozapine require polypharmacy to better stabilize their condition. Such co prescriptions may carry the risk of inducing more side effects especially blood dyscrasias. In our study, we did not find a significant association between psychotropic medication added to clozapine and hematological abnormalities. But further research is warranted to better explore this association.

**Disclosure:**

No significant relationships.

